# A Real-World Observation of Antipsychotic Effects on Brain Volumes and Intrinsic Brain Activity in Schizophrenia

**DOI:** 10.3389/fnins.2021.749316

**Published:** 2022-02-09

**Authors:** Yifan Chen, Fay Y. Womer, Ruiqi Feng, Xizhe Zhang, Yanbo Zhang, Jia Duan, Miao Chang, Zhiyang Yin, Xiaowei Jiang, Shengnan Wei, Yange Wei, Yanqing Tang, Fei Wang

**Affiliations:** ^1^Department of Psychiatry, The First Affiliated Hospital of China Medical University, Shenyang, China; ^2^Early Intervention Unit, Department of Psychiatry, Affiliated Nanjing Brain Hospital, Nanjing Medical University, Nanjing, China; ^3^Department of Radiology, The First Affiliated Hospital of China Medical University, Shenyang, China; ^4^Department of Psychiatry, Washington University School of Medicine, St. Louis, MO, United States; ^5^School of Biomedical Engineering and Informatics, Nanjing Medical University, Nanjing, China; ^6^Nanjing Brain Hospital, Nanjing Medical University, Nanjing, China; ^7^Functional Brain Imaging Institute of Nanjing Medical University, Nanjing, China

**Keywords:** schizophrenia, gray matter volumes, amplitude of low frequency fluctuations, antipsychotics, real-world observation

## Abstract

**Background:**

The confounding effects of antipsychotics that led to the inconsistencies of neuroimaging findings have long been the barriers to understanding the pathophysiology of schizophrenia (SZ). Although it is widely accepted that antipsychotics can alleviate psychotic symptoms during the early most acute phase, the longer-term effects of antipsychotics on the brain have been unclear. This study aims to look at the susceptibility of different imaging measures to longer-term medicated status through real-world observation.

**Methods:**

We compared gray matter volume (GMV) with amplitude of low-frequency fluctuations (ALFFs) in 89 medicated-schizophrenia (med-SZ), 81 unmedicated-schizophrenia (unmed-SZ), and 235 healthy controls (HC), and the differences were explored for relationships between imaging modalities and clinical variables. We also analyzed age-related effects on GMV and ALFF values in the two patient groups (med-SZ and unmed-SZ).

**Results:**

Med-SZ demonstrated less GMV in the prefrontal cortex, temporal lobe, cingulate gyri, and left insula than unmed-SZ and HC (*p* < 0.05, family-wise error corrected). Additionally, GMV loss correlated with psychiatric symptom relief in all SZ. However, medicated status did not influence ALFF values: all SZ showed increased ALFF in the anterior cerebrum and decreased ALFF in posterior visual cortices compared with HC (*p* < 0.05, family-wise error corrected). Age-related GMV effects were seen in all regions, which showed group-level differences except fusiform gyrus. No significant correlation was found between ALFF values and psychiatric symptoms.

**Conclusion:**

GMV loss appeared to be pronounced to longer-term antipsychotics, whereby imbalanced alterations in regional low-frequency fluctuations persisted unaffected by antipsychotic treatment. Our findings may help to understand the disease course of SZ and potentially identify a reliable neuroimaging feature for diagnosis.

## Introduction

Psychotropic medications have long confounded findings from neuroimaging studies. Their effects result in inconsistent findings and hamper efforts to understand further the pathophysiology of neuropsychiatric disorders such as schizophrenia (SZ). Mounting neuroimaging studies have indicated SZ that features structural deficits spanning the multiple brain regions such as frontal, temporal, and parietal lobes (Honea et al., 2005; Cronenwett and Csernansky, 2010; Yang et al., 2021), accompanied by functional abnormalities (Lawrie et al., 2002; Zhou et al., 2007). Despite the availability of various magnetic resonance imaging (MRI) techniques, the consistency of findings across studies remains a significant challenge. Previous studies have demonstrated gray matter volume (GMV) decreased in SZ by 2% (Wright et al., 2000; Vita et al., 2012). However, Benedicto reported no differences in GMV between SZ and healthy volunteers (Kim et al., 2000). Besides, two studies have suggested abnormally increased GMV in the orbitofrontal cortex (Hoptman et al., 2005; Lacerda et al., 2007), whereas a third study detected the opposite change in the same region (Nakamura et al., 2008). Furthermore, a recent selective meta-analysis concluded that the variability of GMV in specific areas such as the putamen and temporal lobe was significantly greater in patients (Brugger and Howes, 2017).

Scholars have emphasized more extensive brain network dysfunction. However, the specific pathological mechanism is not clear, and the high heterogeneity between the studies has also led to more controversy and discussion. For SZ with antipsychotics, there are few studies of structural imaging combined with functional imaging. One of the commonly used functional MRI techniques, such as amplitude of low-frequency fluctuations (ALFF), has been developed, which measures the intensity of spontaneous fluctuation in a particular voxel within the BOLD signal (Zang et al., 2007). The intrinsic brain activity consumes over 95% of the brain’s energy and is likely to play a critical role in brain function (Zhang and Raichle, 2010). A number of recent studies have revealed synchronous changes in brain volume and ALFF values in specific disease processes, suggesting that the two brain measures are related. It is most reliable in gray matter (GM), suggesting that ALFF derives from neural activity (Vesa et al., 2003). Studies have revealed synchronous changes in brain volume and ALFF values in specific disease processes, suggesting that the two brain measures are related (Li et al., 2014; Liu et al., 2014). Notably, Qing and Gong (2016) provide direct empirical evidence of a strong association between brain size/volume and intrinsic brain activity, suggesting that brain size serves as a structural substrate for the intrinsic brain activity of the human brain.

However, studies of ALFF in SZ also have given inconsistent results. A recent meta-analysis in SZ indicated the decreased ALFF primarily in the somatosensory cortex, posterior parietal cortex, and occipital cortex and increased ALFF mainly in the bilateral medial temporal cortex and medial prefrontal cortex (Xu et al., 2015). However, some studies have contrarily reported decreased ALFF in medial prefrontal areas (Huang et al., 2010; Wenting et al., 2013). Similarly, two studies have suggested abnormally high intrinsic activity in the bilateral putamen (Huang et al., 2010; He et al., 2013); Lui did not report such high activity though (Lui et al., 2010). Altogether, although multiple factors could contribute to inconsistent findings, such as differences in sample characteristics or exact methodology, the inconsistencies across studies may, in part, reflect the effects of antipsychotics in SZ.

What the role antipsychotic treatment played on the pathophysiologic process of brain deficits in SZ remains unclear. Antipsychotics have been associated with GMV deficits in the frontotemporal cortex and the whole brain in SZ (Leung et al., 2011; Ahmed et al., 2015) but have also been associated with no change or increase in GMV (Halim et al., 2004; Ho et al., 2011). Also, a meta-analysis indicated that more significant volumetric variation in the brain structure of individuals with SZ compared with controls could suggest that antipsychotic levels may somehow be associated with greater heterogeneity in brain volume in SZ (Kuo and Pogue-Geile, 2019). However, there are still relatively limited studies regarding the influence of medications in ALFF. Six-week antipsychotic treatment has been associated with increases of ALFF in the frontal gyrus, parietal lobule, temporal gyrus, and right caudate (Lui et al., 2010). Furthermore, after 1-year treatment of antipsychotics, the majority of ALFF values did not significantly change between baseline and follow-up imaging except right inferior parietal lobule and orbitofrontal cortex and right occipital gyrus (Li et al., 2016). The earlier findings suggest that neuroimaging measures vary in their susceptibility to medication effects and that some measures more robustly reflect disease processes *versus* treatment response.

Furthermore, although experts agree on the efficacy of antipsychotics in alleviating psychotic symptoms during the early most acute phase (0–2 months), the longer-term effects of antipsychotics on the brain have long been controversial (Vita et al., 2015; van Erp et al., 2018; Liu et al., 2020). Given that evidence for the benefits of antipsychotics extends only to approximately 0–2 years, what to do in the longer term draws our attention. One recent naturalistic longitudinal study of patients with psychosis with a 3-year follow-up period reported a significantly increased rate of cortical loss compared with healthy controls (HCs; Akudjedu et al., 2020). However, infrequent studies assessed how the brain of patients with SZ changes when treated for midterm to long term with antipsychotic medications. Moreover, views about the longer-term (>2 months) efficacy of antipsychotics often based on the results from longitudinal evaluations are subject to the risk of bias caused by dropout or statistical effectiveness reduction. For this reason, the cross-sectional studies combining structural and functional imaging based on the natural world can bring complementary information for brain changes at this stage.

Therefore, in this study, we examined GMV and ALFF using voxel-based morphometry and resting-state functional magnetic resonance imaging (fMRI) techniques in SZ based on medication status: unmedicated (medication naïve or not taking medications for at least 4 weeks before scan) and medicated (taking medications for at least 8 weeks before the scan, more than half of them for 2–4 years). We aimed for a preliminary real-world observation with no study-related and longer-term treatment changes to capture the typical conditions of neuroimaging studies to provide some basis for the identification of objective image indicators in patients with SZ. Thresholds for medication status were determined based on prior findings of antipsychotic effects on the brain as early as 1 month after treatment initiation in SZ and standards for unmedicated status by other studies in the literature (Mcclure et al., 2008; Leung et al., 2011; Kraguljac et al., 2016). We also examined associations between structural and functional metrics and clinical markers in patients with SZ.

## Materials and Methods

### Subjects

The Institutional Review Board of China Medical University approved this study, and all participants provided written informed consent after a detailed description of the study. Participants in this study included 89 individuals with SZ who had been taking only atypical antipsychotics for more than 8 weeks before the time of scan (med-SZ) and 81 individuals with SZ (unmed-SZ) who were either antipsychotics naïve (*n* = 31, 38.27%) or not taking antipsychotics for at least 4 weeks at the time of scan (*n* = 50, 61.73%) and 235 HCs aged 15–55 years. We reviewed information on the type and dosage of medication recorded at the time of the MRI scan. Med-SZ was treated with antipsychotics including clozapine, risperidone, olanzapine, aripiprazole and quetiapine, aripiprazole, and ziprasidone. The doses of antipsychotics were converted to olanzapine equivalents based on defined daily doses (Leucht et al., 2016). All participants were recruited from the outpatient clinics of the Department of Psychiatry, The First Affiliated Hospital of China Medical University, Shenyang, China, and HC participants were recruited from Shenyang, China, by advertisement. The absence or presence of Axis I disorders was independently assessed by two trained psychiatrists using the Structured Clinical Interview for Diagnostic and Statistical Manual of Mental Disorders, Fourth Edition, Axis I Disorders for participants older than 18 years and the Schedule for Affective Disorders and Schizophrenia for School-Age Children—Present and Lifetime Version (K-SADS-PL) for participants younger than 18 years. SZ participants met the Diagnostic and Statistical Manual of Mental Disorders, Fourth Edition, diagnostic criteria for SZ, schizophreniform disorder, schizoaffective disorder, and no other Axis I disorder. HC participants did not have current or lifetime Axis I disorder or history of psychotic, mood, or other Axis I disorders in first-degree relatives (as determined from detailed family history). For all participant groups, individuals were excluded for the history of substance or alcohol abuse or dependence, head injury, neurologic or concomitant major medical disorder, and any MRI contraindications for MRI symptom measures using the Brief Psychiatric Rating Scale (BPRS) in patients with SZ. Table 1 outlines the participants’ demographic and clinical status at the time of testing.

**TABLE 1:** Demographic and clinical characteristics of healthy controls, medicated schizophrenia, and unmedicated schizophrenia.

HC (*n* = 235)		SZ (*n* = 170)		*F/*χ*^2^/t*-values	*P*-values
		Med-SZ (*n* = 89)	Unmed-SZ (*n* = 81)		
**Demographic characteristic**					
Age at scan, years	28.17 (0.53)	27.65 (10.06)	25.53 (9.30)	2.740^a^	0.066
Age distribution					
−15 to 28	161 (68.51%)	53 (59.55%)	53 (65.43%)		
−29 to 42	54 (22.98%)	24 (26.97%)	22 (27.16%)		
−43 to 55	20 (8.51%)	12 (13.48%)	6 (7.40%)		
Sex (male:female)	93 (39.57%)	44 (49.44%)	32 (43.20%)	2.789^a^	0.248
Right handedness	217 (92.34%)	78 (87.64%)	68 (83.95%)	2.525^a^	0.640
Education	15.08 (3.06)	12.06 (2.98)	10.93 (3.00)	70.832	<0.001
**Clinical characteristic**					
First episode, yes	\	44 (49.44%)	71 (87.65%)	\	<0.001
Illness duration (months)	\	43.07 (51.01)	8.84 (17.83)	31.14^b^	<0.001
Medication use^c^	\				
Durations (months)		12.34 (13.74)	\		
-Olanzapine		21			
-Clozapine		13			
-Risperidone		41			
-Aripiprazole		19			
-Ziprasidone		4			
-Quetiapine		5			
Olanzapine equivalents		3.93 (6.15)			
BPRS-Factors	*n* = 159	*n* = 83	*n* = 77		
Anxiety and depression	4.28 (0.83)	6.89 (3.72)	8.90 (4.35)	5.342^b^	0.009
Lack of energy	4.03 (0.21)	6.53 (3.03)	8.14 (4.54)	11.35^b^	<0.001
Thought disorder	4 (0)	6.65 (3.77)	9.39 (4.89)	6.844^b^	0.176
Activity	3.06 (0.26)	4.29 (2.09)	4.78 (2.47)	3.267^b^	<0.001
Hostility	3.02 (0.14)	4.61 (2.78)	7.44 (4.01)	18.19^b^	<0.001
BPRS-Total score	18.39 (1.21)	28.98 (10.30)	39.03 (14.62)	9.042^b^	0.002

*HC, healthy controls; med-SZ, medicated schizophrenia; unmed-SZ, unmedicated schizophrenia; BPRS, Brief Psychiatric Rating Scale. Data are n (%) or mean (SD).*

*^a^Examination among healthy control, medicated schizophrenia, and unmedicated schizophrenia groups.*

*^b^Examination between medicated schizophrenia and unmedicated schizophrenia groups.*

*^c^This refers to medication patients were prescribed on day of their MRI scan and not to their cumulative lifetime exposure.*

### Image Acquisition

The scan was performed using a 3T MRI scanner (General Electric, Milwaukee, WI, United States) at the Image Institute of The First Affiliated Hospital of China Medical University, Shenyang, China. The three-dimensional fast spoiled gradient-echo sequence was used to obtain sagittal T1-weighted structural images of the whole brain. Scanning parameters were as follows: repetition time = 7.1 ms, echo time = 3.2 ms, field of view = 24 cm × 24 cm, voxel size = 1 mm × 1 mm × 1 mm, slice thickness = 1.0 mm without a gap, 176 slices in total, and the scan time = 8 min 6 s. Functional images were collected using a gradient echo-planar imaging sequence: repetition time = 2,000 ms, echo time = 30 ms, field of view = 240 cm × 240 cm, flip angle = 90°, matrix = 64 × 64, and slices = 35. Participants were instructed to rest with their eyes closed but remain awake during scanning.

### Structural Magnetic Resonance Imaging Data Processing

Processing was performed using the DARTEL algorithm Statistical Parametric Mapping software (SPM8)^1^ under the MATLAB R2010b platform (MathWorks, Sherborn, MA, United States). Briefly, the segmentation function was used to divide the regions into GM, white matter, and cerebrospinal fluid using the “new segment” tool implemented in SPM8. During spatial normalization, inter-subject registration was achieved using separate registration based on group assignment. A modulation step was used to ensure that the overall amount of tissue in a class was unaltered. The segmented images were normalized to the Montreal Neurological Institute template and were smoothed with an 8-mm full width at a half-maximum Gaussian filter. The voxel size of data acquisition was 1 mm^3^, and the voxel size of normalized data was 1.5 mm^3^.

### Functional Magnetic Resonance Image Processing and Amplitude of Low-Frequency Fluctuation Calculation

Resting-state fMRI data preprocessing was carried out using Statistical Parametric Mapping 8 (SPM)^2^. Data Processing Assistant for Resting-State fMRI (DPARSF, V2.0_101025)^3^ was based on SPM8. For each participant, the first 10 volumes of scanned data were discarded to allow for steady-state magnetization. Further data preprocessing included slice timing correction and head motion correction. Each participant’s motion was assessed by means of translation/rotation and an exclusion criterion (translation >3 mm, rotation >3° in each participant). Then, spatial normalization was performed using the standard echo-planar imaging template from the Montreal Neurological Institute Spatial smoothing, which was done with a 6-mm full width at a half-maximum Gaussian filter, and linear detrending was performed to remove linear trends (Cordes et al., 2001). The filtered time series was transformed into the frequency domain with a fast Fourier transform, and the power spectrum was then obtained. ALFF was measured by obtaining the square root of the signal across 0.01–0.08 Hz for each voxel.

### Statistical Analysis

Analyses of demographic and clinical characteristics were performed using analysis of variance and chi-square tests. Results were considered significant at *p* < 0.05. To compare GMV with ALFF values among the med-SZ, unmed-SZ, and HC groups, whole brain GMV and ALFF values comparisons among three groups were performed using a full-factorial design, with age and sex as covariates, using SPM8 (Wellcome Trust Centre for Neuroimaging, see text footnote 1). The GMV and ALFF value differences were considered significant at a height threshold of *p* < 0.05, family-wise error (FWE) corrected for multiple comparisons, and an extended threshold of 20 voxels (Liao et al., 2015). GMV and ALFF values were extracted from all significant regions in the three-group analysis and analyzed in *post hoc* pair-wise t-contrasts (med-SZ *vs*. unmed-SZ, med-SZ *vs*. HC, and unmed-SZ *vs*. HC) using SPSS. Duration of illness was included as a covariate of non-interest in the patient subgroup comparison. To determine age-related effects on GMV and ALFF values in the two patient groups (medicated and unmedicated), we used the mean values of GMV and ALFF in regions showing the group-level difference and ran a general linear model in SPSS. The GMV and ALFF values were considered as dependent variables. Fixed factors included age and group (med-SZ and unmed-SZ). Analyses were also performed with false discovery rate (FDR) corrected for multiple comparisons to further confirm the findings of age-related effects.

We used exploratory Pearson’s correlation analyses to examine the relationship between GMV/ALFF values and BPRS scores and medication time in each patient group. Analyses were also performed with FDR corrected for multiple comparisons to further confirm the findings of correlation after FDR correction; *p* < 0.005 was considered significant in correlation analysis.

## Results

### Demographic and Clinical Data

There was no significant difference among med-SZ, unmed-SZ, and HC groups in age (*p* = 0.066) or sex (*p* = 0.248), and handedness (*p* = 0.640). The unmed-SZ group had significantly shorter illness duration, lower first-episode rate, and higher BPRS scores compared with the med-SZ group (see Table 1 for details).

### Gray Matter Volume Findings

**FIGURE 1:**
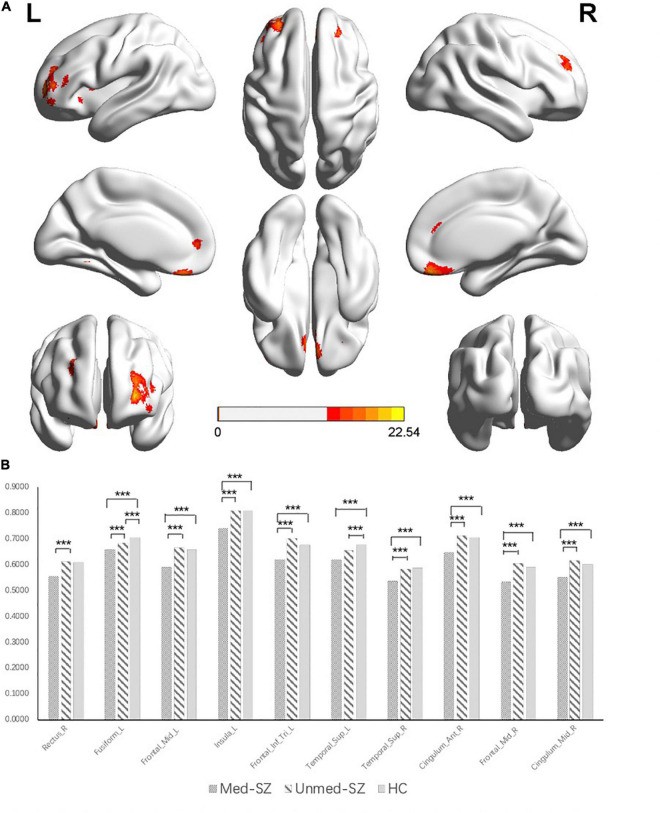
**(A)** Significant differences in gray matter volumes among medicated schizophrenia, unmedicated schizophrenia, and healthy controls. ***Significant at *P* < 0.05 corrected by FWE correction. **(B)** Gray matter volumes in regions showing significant differences among medicated schizophrenia, unmedicated schizophrenia, and healthy controls. R, right; L, left; Frontal_Mid, middle frontal gyrus; Frontal_Inf_Tri, Inferior Pars Triangularis; Temporal_Sup, superior temporal cortex; Cingulum_Ant, anterior cingulate cortex; Cingulum_Mid, middle cingulate cortex.

Significant group differences were found in several regions consisting of the prefrontal cortex, temporal lobe, cingulate gyri, and insula (*df* = 2, *p* < 0.05, FWE corrected) (Figure 1 and Table 2). Compared with unmed-SZ and HC groups, the med-SZ demonstrated decreased GMVs located in bilateral middle frontal gyrus, bilateral superior medial frontal gyrus, bilateral superior temporal gyrus, bilateral anterior cingulate gyri, bilateral middle cingulate gyri, right Rolandic operculum, right superior frontal gyrus, middle orbital frontal gyrus, left fusiform gyrus, and left insula. All clusters except the left superior temporal cortex survived when illness duration was included as a covariate in comparison between med-SZ and unmed-SZ. Significant decreases were found in the left fusiform gyrus and left superior temporal gyrus in unmed-SZ compared with HC (Figure 1 and Table 2).

**TABLE 2:** Regions with GM differences in demonstrating significant group differences.

Cortical regions	Cluster size	MNI coordinates			*F* values*
		X	Y	Z	
A R rectus gyrus	1,873	−2	50	3	22.5416
L superior medial frontal gyrus					
L rectus gyrus					
R superior medial frontal gyrus					
L anterior cingulum					
B L fusiform gyrus	43	−33	−53	−11	15.7687
C L middle frontal gyrus	1,011	−33	48	6	21.3453
L middle orbital frontal gyrus					
D L insula	30	−35	11	6	14.5635
E L inferior pars triangularis	79	−47	38	12	15.7309
F L superior temporal gyrus	43	−56	−14	9	17.1504
G R superior temporal gyrus	78	63	−17	12	15.953
R Rolandic operculum					
H R anterior cingulum	57	8	36	23	14.356
I R middle frontal gyrus	314	32	54	23	19.4355
R superior frontal gyrus					
J R middle cingulum	109	2	15	36	17.987
L middle cingulum					

*MNI, Montreal Neurological Institute; L, Left; R, Right.*

*Significant at P < 0.05 corrected by FWE correction.

### Amplitude of Low-Frequency Fluctuation Findings

**FIGURE 2:**
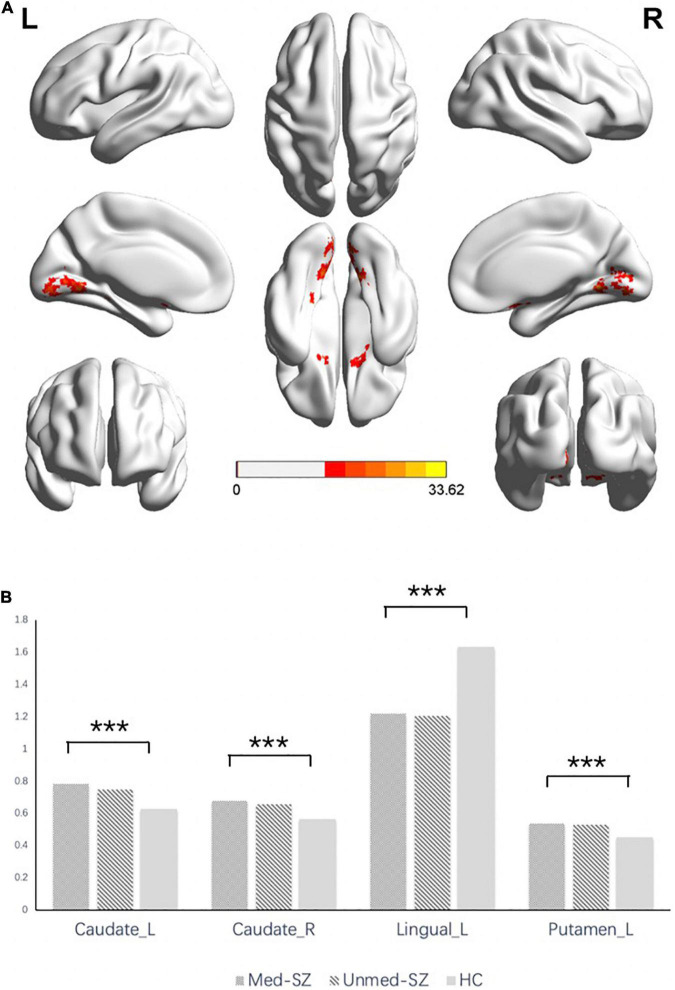
**(A)** Significant differences in ALFF among medicated schizophrenia, unmedicated schizophrenia, and healthy controls. ***Significant at *P* < 0.05 corrected by FWE correction. **(B)** ALFF values in regions showing significant differences among medicated schizophrenia, unmedicated schizophrenia, and healthy controls. R, right; L, left; Caudate, caudate nucleus; Lingual, lingual gyrus.

The three-group analysis of ALFF found four clusters showing significant group differences (*df* = 2, *p* < 0.05, FWE corrected). These clusters are situated in the bilateral occipital lobe, bilateral striatum, and left parahippocampal gyrus (Figure 2 and Table 3). Compared with HC, med-SZ and unmed-SZ had significant decreases of ALFF values in bilateral lingual and calcarine gyrus and significant increases mainly in bilateral caudate and putamen, as well as left parahippocampal gyrus. No differences were observed between med-SZ and unmed-SZ (Figure 2 and Table 3).

**TABLE 3:** Regions with ALFF differences in demonstrating significant group differences.

Cortical regions	Cluster size	MNI coordinates			*F* values*
		X	Y	Z	
A L caudate	100	−18	21	−3	33.6157
B R caudate	137	15	12	−15	24.0428
R putamen					
C L lingual gyrus	317	−15	−63	−3	24.2724
R lingual gyrus					
L calcarine					
R calcarine					
D L putamen	22	−24	−6	9	24.3612

*MNI, Montreal Neurological Institute; L, Left; R, Right.*

*Significant at P < 0.05 corrected by FWE correction.

### Age-Related Differences in Gray Matter Volume and Amplitude of Low-Frequency Fluctuation

We examined relationships between age and GMV that differed between the two groups. More specifically, age-related GMV effects were seen in all regions, which showed group-level differences except fusiform gyrus. *Post hoc* analyses showed that the age-related decline in GMV of the bilateral middle frontal gyrus, left inferior pars triangularis, and right middle cingulum in medicated patients was significantly greater than that in unmedicated patients. Unmedicated patients showed significantly faster age-related GMV decline in the right rectus gyrus, left insula, bilateral superior temporal gyrus, and right anterior cingulum than medicated subjects. We also examined relationships between age and ALFF values that differed between the two groups but found no association after FDR correction.

### Correlations With Clinical Variables and Medication Time

For both med-SZ and unmed-SZ, BPRS scores were positively correlated with GMV in bilateral middle frontal gyrus, bilateral superior medial frontal gyrus, right superior temporal gyrus, bilateral anterior cingulate gyri, bilateral middle cingulate gyri, right Rolandic operculum, right superior frontal gyrus, middle orbital frontal gyrus, and left insula. After FDR correction, most correlations survived, except the correlation between BPRS scores and the GMV in left fusiform and left superior temporal gyrus (*p* < 0.05, FDR corrected) (Supplementary Table 1 and Table 2). No significant correlation was found between ALFF values and BPRS scores (Supplementary Table 2 and Table 3). Besides, we did not detect any relation between medication time and GMV/ALFF values (Supplementary Tables 1, 2 and Tables 2, 3).

## Discussion

In the current study, we compared the susceptibility of different imaging measures with antipsychotics. We found significant GMV decreases in the prefrontal cortex, temporal lobe, cingulate gyri, and left insula in med-SZ compared with unmed-SZ and HC. Among these regions, unmed-SZ demonstrated lower GMV in the left superior temporal cortex and left fusiform than HC additionally. We also found a significant correlation between GMV loss and decline of BPRS scores. No significant differences in ALFF were found based on medication status, and no significant correlations between ALFF and BPRS scores were observed. Both med- and unmed-SZ had significantly increased ALFF in the anterior cerebrum, including bilateral striatum and left parahippocampal, and decreased ALFF in posterior visual cortices, including the calcarine and lingual gyrus, compared with HC. Our findings indicate that GMV may be susceptible to medication effects, whereas ALFF is not in SZ.

It is widely accepted that SZ is characterized by extensive GMV abnormalities (Kubicki et al., 2002; Koolschijn et al., 2010; Pinacamacho et al., 2016); however, the contribution of medication effects to these alterations are unclear. Moreover, further investigation is needed to clarify if specific regions are more affected by neuroleptics. Our findings suggest that antipsychotic treatment may be more influential in GMV in the frontal, temporal, cingulate, and insula regions. This result is in line with evidence from substantial imaging studies indicating that antipsychotic treatment can result in GMV loss in SZ (Mccormick et al., 2005; Ho et al., 2011). Tyler et al. reported that significant cortical thinning was identified in the medicated patient group relative to the control group in prefrontal, temporal, parietal, and occipital cortices (Lesh et al., 2015). The unmedicated patient group showed no significant cortical thickness differences from the control group. Furthermore, meta-regression analyses also reported that GMV in medial frontal, anterior cingulate cortices, and left insular regions were more decreased in med-SZ (Radua et al., 2012). A study of 211 individuals with SZ over a mean 7.2-year period found that antipsychotics significantly influenced brain volumes even after accounting for illness duration, illness severity, and substance use (Ho et al., 2011). Antipsychotic dosing explained 1.7% of the decreases in total GMV (Ho et al., 2011). Animal studies also support the earlier findings that non-human primates given doses of antipsychotics similar to those given to humans showed brain volume reductions of around 10%, mostly attributable to loss of the glial cells that support and protect neurons (Cyranoski, 2011). Although substantial findings, including our results, suggest GMV loss is induced by antipsychotics (Lieberman et al., 2005; Radua et al., 2012), a few findings have identified conserved or even increased GMV in SZ with specific atypical antipsychotics (Gur et al., 1998; Molina et al., 2005), and that may be attributed to differences in sample size, the definition of medicated and unmedicated groups: in many of these studies, the sample sizes were modest (<30 subjects); the treatment duration is relatively short (mostly <6 weeks).

Gray matter volume changes in SZ do not appear entirely due to medication effects. We found significant GMV loss in the left superior temporal cortex and left fusiform in unmed-SZ, compared with HC. Previous studies have shown GMV reduction in left posterior superior temporal gyrus and left fusiform in first-episode SZ (Hirayasu et al., 1998; Lee et al., 2002, 2015). Our findings replicate prior findings of decreased left fusiform volume in treatment-naive SZ (Guo et al., 2013). Structural alterations in the left superior temporal cortex and left fusiform structural abnormalities appear less affected by illness chronicity and medication effects and hence may serve as potential biomarkers for SZ.

Gray matter volumes in prefrontal, temporal, cingulate, and insula were positively correlated with BPRS scores, consistent with previous findings (Wenting et al., 2013; Liao et al., 2015). We found GMV deficits found in frontotemporal regions that have been implicated in aggression, emotional withdrawal, blunted affect, and conceptual disorganization in SZ (Lieberman et al., 2005; Cobia et al., 2012; Fusarpoli et al., 2013). The correlation between GMV deficits and BPRS scores may reflect GMV as a marker of disease state (*versus* trait) and treatment response.

We found age-related differences in GMV between med- and unmed-SZ. More specifically, in patients with med-SZ, imaging studies found accelerated age-related GMV decline than unmed-SZ in all regions showing group-level differences except the fusiform gyrus. To our best knowledge, this is the first cross-sectional study that explores the relation between GMV and age with or without longer medication use. This finding is also consistent with previous studies suggesting that progressive brain GMV changes during the life-long course of SZ appeared in part to be related to antipsychotics (Ho et al., 2011; Lesh et al., 2015). Moreover, a recent randomized, double-blind placebo-controlled clinical trial design study found a significant treatment-group × time interaction in relation to cortical thickness with 36-week treatment (Voineskos et al., 2020). Given that medication use may bring out a detrimental effect of progressive GMV changes, which are typically interpreted in psychiatric and neurologic disorders as non-desirable, our findings could support a reconsideration of the risks and benefits of antipsychotics.

Here, we provided evidence focused on the longer-term antipsychotic treatment showing that we may underestimate the risks of longer-term antipsychotic treatment. The temporal relief of symptoms may be a compensation for GMV loss; the coverage of the worrying issue of the longer-term effects of antipsychotics on brain structure loss must not be overlooked. Recently, Correll et al. (2018) argued for a positive view of the risk–benefit ratio for long-term continuous antipsychotic treatment of SZ. However, Correll et al. (2018) too readily dismiss the evidence that prolonged antipsychotic use is associated with decreased GM and fail to cite the monkey and rodent studies in which administration of antipsychotics causes brain volume losses (Cyranoski, 2011). As we have noted, the association between cumulative dose of antipsychotics and general GMV reduction has been repeatedly reported. Moreover, the accompanying dopamine hypersensitivity psychosis and poor long-term (7–20 years) prognosis suggest the inevitable issue of its adverse effects on brain structure (Martin and Jobe, 2018). Furthermore, some studies assess whether patients with SZ improve when treated longer than 2 or 3 years with antipsychotic medications (Morgan et al., 2014; Harrow et al., 2017; Velthorst et al., 2017). Interestingly, unlike short-term studies, none of them showed positive long-term results. Although administered widely, we still do not have a clear knowledge of all of the antipsychotic effects. Further questions and studies of it would seem critical.

Our findings with ALFF suggest that ALFF is less influenced by medication and disease state. We found increased ALFF in the anterior cerebrum, including the bilateral striatum, and decreased ALFF in the posterior visual cortex, consistent with previous studies in both unmed- and med-SZ (He et al., 2013; Li et al., 2017; Chang et al., 2019). A meta-analysis also concluded that the foci with increased ALFF are mainly located in the striatum and decreased ALFF situated in the occipital cortex consistently (Xu et al., 2015). The striatum, together with other basal ganglia nuclei, regulates information flow to and from the cerebral cortex and other related subcortical regions (such as the thalamus). Our finding of altered ALFF in the bilateral striatum is consistent with prior studies implicating striatal abnormalities in the pathophysiology of SZ. Prior functional and metabolic studies have found increased striatal dopamine neurotransmission and activity in SZ (Kegeles et al., 2010; Zhuo et al., 2014), and our results may support the hypothesis that the striatum of SZ is in a state of hyper-dopamine (Abidargham, 2014).

Bilateral lingual and calcarine gyrus, which function as the primary visual cortex, have been related to visual hallucinations in most studies (González-Hernández et al., 2003; Onitsuka et al., 2007; Hoptman et al., 2010; Lang et al., 2010), suggesting that the visual cortex alterations may be important in SZ. We found that med-SZ and unmed-SZ showed decreased ALFF values in the posterior visual cortex, including lingual gyrus and calcarine gyrus, consistent with the hypothesis that SZ is associated with low-level sensory functions (Butler et al., 2001; Butler and Javitt, 2005). For instance, the magnocellular deficits were found in SZ, which may lead to their higher-level visual processing difficulties as well (Butler and Javitt, 2005). However, a previous functional neuroimaging study reported a different result from ours. Increased ALFF was found in the bilateral prefrontal and parietal cortex, left superior temporal cortex, and right caudate nucleus after 6-week treatment of atypical antipsychotics (Lui et al., 2010), but this finding has not been replicated, and the heterogeneity of findings may be caused by sample differences such as illness chronicity. The ALFF signal is correlated with baseline cerebral blood flow and is thought to reflect spontaneous, intrinsic neuronal activity (Zhou et al., 2010). ALFF signal is mostly reliable in GM, not white matter, suggesting that ALFF derives from neural activity, although further work is needed for the exact neural substrate for ALFF (Kiviniemi et al., 2003). Our studies suggest that these abnormalities may be trait-like alterations of brain function that current antipsychotics do not effectively normalize.

No significant correlation was found between ALFF and BPRS, indicating that amplitude cannot be used as a quantitative marker for the assessment of symptoms of SZ, although it can be used in a qualitative way to help locate aberrant functional areas. Previous studies have shown that striatal ALFF was not associated with psychotic symptom severity in the first episode and medication naïve SZ (Huang et al., 2010; He et al., 2013; Wenting et al., 2013; Li et al., 2017). Anna et al. reported that SZ exhibited decreased ALFF values in the occipital pole and lingual gyrus regardless of the severity of auditory verbal hallucinations (Alonso-Solís et al., 2017). Thus, ALFF appears to be a relatively static measure that does not vary with state changes in SZ.

There are several limitations to this study. As this represented a real-world observational study, data regarding medication dose, compliance, and duration were not available or included in our analyses. Besides, the ratio of antipsychotic-naïve in the unmed-SZ was 38.3%, which may be a potential confounding effect of medication exposure in the unmed-SZ group. Furthermore, the study was cross-sectional and not longitudinal, in which the same individual was observed in medicated and unmedicated state, although the demographic data among the three groups have been matched to reduce the influence of confounding factors to a certain extent. Thus, further research is needed to confirm the generalizability of our findings.

Through real-world observation, our study suggests that GMV is a sensitive spatiotemporal marker of longer-term pharmacologic intervention in the brain, whereas ALFF is a relatively static measure reflecting more core disease traits that are less influenced by medication status and disease state (e.g., active *versus* remitted). Our study underscores the need for careful selection of neuroimaging measures based on study purpose and the focus on longer-term antipsychotics (e.g., use GMV when primary interest in treatment response and ALFF when main focus on disease trait).

## Data Availability Statement

The original contributions presented in the study are included in the article/Supplementary Material, further inquiries can be directed to the corresponding authors.

## Ethics Statement

The studies involving human participants were reviewed and approved by the Medical Science Research Ethics Committee of The First Affiliated Hospital of China Medical University. Written informed consent to participate in this study was provided by the participants’ legal guardian/next of kin.

## Author Contributions

FW and YT designed the experiments. YC, MC, RF, JD, ZY, YW, SW, and XJ acquired the data. YC, RF, and XZ analyzed the data. YC and FW wrote the manuscript. FYW and FW reviewed or edited the manuscript. All authors discussed the results, reviewed the manuscript, and meet the criteria for authorship to be named as authors.

## Conflict of Interest

The authors declare that the research was conducted in the absence of any commercial or financial relationships that could be construed as a potential conflict of interest.

## Publisher’s Note

All claims expressed in this article are solely those of the authors and do not necessarily represent those of their affiliated organizations, or those of the publisher, the editors and the reviewers. Any product that may be evaluated in this article, or claim that may be made by its manufacturer, is not guaranteed or endorsed by the publisher.

## Footnotes

1
http://www.fil.ion.ucl.ac.uk/spm/software/spm8/

2
http://www.fil.ion.ucl.ac.uk/spm

3
http://www.restfmri.net
