# Supplementary material for: A Real-World Observation of Antipsychotic Effects on Brain Volumes and Intrinsic Brain Activity in Schizophrenia
Source: Front Neurosci. 2022 Feb 9;15:749316. doi: 10.3389/fnins.2021.749316 (PMC8863862; doi:10.3389/fnins.2021.749316)
Supplement: Supplementary file 1 [file Data_Sheet_1.docx]

**TableS-1. Correlation between GMV and clinical factors**

| **Clinical variables** | | **CLA** | **CLB** | **CLC** | **CLD** | **CLE** | **CLF** | **CLG** | **CLH** | **CLI** | **CLJ** |
| --- | --- | --- | --- | --- | --- | --- | --- | --- | --- | --- | --- |
| BPRS Total Scores | *r-value* | .308 | .047 | .281 | .270 | .235 | .175 | .238 | .259 | .262 | .316 |
|  | *p-value* | <.001 | .585 | .001 | .001 | .006 | .041 | .005 | .002 | .002 | <.001 |
| Anxiety and depression | *r-value* | .073 | -.016 | .031 | .143 | .021 | .006 | .078 | .104 | .028 | .110 |
|  | *p-value* | .397 | .855 | .720 | .097 | .806 | .943 | .366 | .230 | .746 | .204 |
| Lack of energy | *r-value* | .251 | .015 | .237 | .272 | .190 | .117 | .214 | .235 | .272 | .227 |
|  | *p-value* | .003 | .862 | .005 | .001 | .027 | .176 | .012 | .006 | .001 | .008 |
| Thought disorder | *r-value* | .292 | .063 | .328 | .206 | .224 | .203 | .220 | .259 | .261 | .291 |
|  | *p-value* | .001 | .468 | <.001 | .016 | .009 | .018 | .010 | .002 | .002 | .001 |
| Activity | *r-value* | .189 | .129 | .124 | .059 | .051 | .092 | .078 | .075 | .075 | .201 |
|  | *p-value* | .027 | .135 | .149 | .494 | .557 | .286 | .365 | .383 | .383 | .019 |
| Hostility | *r-value* | .300 | .030 | .272 | .233 | .275 | .185 | .195 | .232 | .227 | .323 |
|  | *p-value* | <.001 | .728 | .001 | .006 | .001 | .031 | .023 | .007 | .008 | <.001 |
| **Medication time** | *r-value*  *p-value* | -.193  .307 | -.075  .692 | -.008  .965 | .006  .974 | .075  .694 | -.093  .625 | -.037  .846 | -.018  .925 | -.041  .829 | -.207  .272 |

*Note:* GMV，grey matter volumes.

BPRS，Brief Psychiatric Rating Scale.

**Table S-2. Correlation between ALFF values and clinical factors**

| **Clinical variables** | | **CLK** | **CLL** | **CLM** | **CLN** |
| --- | --- | --- | --- | --- | --- |
| BPRS Total Scores | *r-value* | .137 | .071 | -.081 | .008 |
|  | *p-value* | .14 | .445 | .383 | .928 |
| Anxiety and depression | *r-value* | .031 | .077 | .035 | -.027 |
|  | *p-value* | .739 | .407 | .706 | .772 |
| Lack of energy | *r-value* | .141 | .097 | -.235 | .084 |
|  | *p-value* | .129 | .3 | .011 | .369 |
| Thought disorder | *r-value* | .211 | .1 | -.091 | .071 |
|  | *p-value* | .022 | .284 | .33 | .45 |
| Activity | *r-value* | .09 | .072 | .036 | -.067 |
|  | *p-value* | .333 | .442 | .699 | .472 |
| Hostility | *r-value* | .067 | -.096 | .017 | -.074 |
|  | *p-value* | .475 | .302 | .854 | .429 |
| **Medication time** | *r-value*  *p-value* | -.011  .963 | .097  .684 | -.253  .281 | -.086  .718 |

*Note: ALFF，*[*Amplitude of Low Frequency Fluctuations*](http://www.baidu.com/link?url=W_n9rFoqYulfKJDHFwzjgblaQ5-Cl2lQwUXjYP2hibEwVuSUHP3emH8QmLnhi-oQMeS4lrbnlzjzELjya1aHia)*.*

*BPRS，Brief Psychiatric Rating Scale.*
